# Targeting low- or high-normal Carbon dioxide, Oxygen, and Mean arterial pressure After Cardiac Arrest and REsuscitation: study protocol for a randomized pilot trial

**DOI:** 10.1186/s13063-017-2257-0

**Published:** 2017-10-30

**Authors:** Pekka Jakkula, Matti Reinikainen, Johanna Hästbacka, Ville Pettilä, Pekka Loisa, Sari Karlsson, Raili Laru-Sompa, Stepani Bendel, Tuomas Oksanen, Thomas Birkelund, Marjaana Tiainen, Jussi Toppila, Antti Hakkarainen, Markus B. Skrifvars, COMACARE study group

**Affiliations:** 1 University of Helsinki and Helsinki University Hospital, Helsinki, Finland; 2 North Karelia Central Hospital, Joensuu, Finland; 3 Päijät-Häme Central Hospital, Lahti, Finland; 4 Tampere University Hospital, Tampere, Finland; 5 Central Finland Central Hospital, Jyväskylä, Finland; 6 Kuopio University Hospital, Kuopio, Finland; 7 Jorvi Hospital, Espoo, Finland; 8 Aarhus University Hospital, Aarhus, Denmark; 9 Australia and New Zealand Intensive Care Research Centre, School of Public Health and Preventive Medicine, Monash University, Melbourne, VIC Australia

**Keywords:** Cardiac arrest, Resuscitation, Carbon dioxide, Oxygen, Blood pressure, Mechanical ventilation, Intensive care

## Abstract

**Background:**

Arterial carbon dioxide tension (PaCO_2_), oxygen tension (PaO_2_), and mean arterial pressure (MAP) are modifiable factors that affect cerebral blood flow (CBF), cerebral oxygen delivery, and potentially the course of brain injury after cardiac arrest. No evidence regarding optimal treatment targets exists.

**Methods:**

The Carbon dioxide, Oxygen, and Mean arterial pressure After Cardiac Arrest and REsuscitation (COMACARE) trial is a pilot multi-center randomized controlled trial (RCT) assessing the feasibility of targeting low- or high-normal PaCO_2_, PaO_2_, and MAP in comatose, mechanically ventilated patients after out-of-hospital cardiac arrest (OHCA), as well as its effect on brain injury markers. Using a 2^3^ factorial design, participants are randomized upon admission to an intensive care unit into one of eight groups with various combinations of PaCO_2_, PaO_2_, and MAP target levels for 36 h after admission.

The primary outcome is neuron-specific enolase (NSE) serum concentration at 48 h after cardiac arrest. The main feasibility outcome is the between-group differences in PaCO2, PaO2, and MAP during the 36 h after ICU admission. Secondary outcomes include serum concentrations of NSE, S100 protein, and cardiac troponin at 24, 48, and 72 h after cardiac arrest; cerebral oxygenation, measured with near-infrared spectroscopy (NIRS); potential differences in epileptic activity, monitored via continuous electroencephalogram (EEG); and neurological outcomes at six months after cardiac arrest.

**Discussion:**

The trial began in March 2016 and participant recruitment has begun in all seven study sites as of March 2017. Currently, 115 of the total of 120 patients have been included. When completed, the results of this trial will provide preliminary clinical evidence regarding the feasibility of targeting low- or high-normal PaCO_2_, PaO_2_, and MAP values and its effect on developing brain injury, brain oxygenation, and epileptic seizures after cardiac arrest. The results of this trial will be used to evaluate whether a larger RCT on this subject is justified.

**Trial registration:**

ClinicalTrials.gov, NCT02698917. Registered on 26 January 2016.

**Electronic supplementary material:**

The online version of this article (doi:10.1186/s13063-017-2257-0) contains supplementary material, which is available to authorized users.

## Background

Hypoxic ischemic encephalopathy is the leading cause of morbidity and mortality after out-of-hospital cardiac arrest (OHCA) [1]. The development of neurological injury occurs during the first 48 h after cardiac arrest, with cerebral hypoperfusion being a potential mechanism [2, 3]. The levels of arterial carbon dioxide tension (PaCO_2_), arterial oxygen tension (PaO_2_), and mean arterial pressure (MAP) may be modified during this period and this may influence cerebral blood flow (CBF), cerebral oxygen delivery, and potentially the severity of the developing brain injury. The optimal targets for PaCO_2_, PaO_2_, and MAP are currently undefined [4].

PaCO_2_ is a major determinant of CBF [5]. After the return of spontaneous circulation (ROSC), the autoregulation of CBF is impaired [6], but reactivity to changes in PaCO_2_ remains [7]. In addition to its role in regulating cerebral perfusion, CO_2_ may have anti-convulsive [8], anti-inflammatory, and anti-oxidant properties [9]. In animal models, moderate hypercapnia (PaCO_2_ 8.0–13.3 kPa) has been related to better neurological scores, less histological brain damage, and less apoptosis compared with normocapnia or severe hypercapnia after cerebral ischemia [10]. Observational studies in humans suggest that hypocapnia is associated with poor outcomes but that mild hypercapnia may be beneficial during the post-resuscitation period after cardiac arrest [11–13]. In the only randomized controlled trial (RCT) on this subject, the investigators compared mild hypercapnia (6.7–7.3 kPa) with normocapnia (4.5–6.0 kPa). The primary outcome was serum neuron-specific enolase (NSE) and S100b protein concentrations over the first 72 h. In this study, mild hypercapnia was associated with significantly lower NSE concentrations compared with normocapnia and no adverse effects were found to be related to higher PaCO_2_ levels [14]. However, hypoventilation and hypercapnia may aggravate acidosis, which is associated with poor neurological outcomes after cardiac arrest [15]. Moreover, in patients with severe acute respiratory distress syndrome (ARDS), acidosis and hypercapnia are associated with impaired right ventricular function and hemodynamic instability, which could impair the recovery of patients with post-cardiac arrest syndrome [16]. Thus far, all studies on this subject have compared hypocapnia and hypercapnia with normocapnia and there are no published data assessing the potential difference between high-normal and low-normal PaCO_2_ values, even though the normal range for PaCO_2_ values is relatively broad (4.5–6.0 kPa).

It has been suggested that exposure to high levels of PaO_2_ during the early stages of reperfusion after cardiac arrest may increase free radical production and thus exacerbate ischemia-reperfusion injury [17]. Retrospective cohort studies have found an association between severe hyperoxia (PaO_2_ > 40 kPa) and poor prognosis after cardiac arrest [18–21]. One observational study with frequent arterial blood gas (ABG) samples found that the combination of moderate hypercarbia and mild hyperoxia was associated with improved neurological recovery [12]. Another recent analysis of a high-resolution cardiac arrest database found that severe hyperoxia (PaO_2_ > 40 kPa) was associated with increased in-hospital mortality, while moderate hyperoxia (13.5–39.9 kPa) was correlated with improved organ function at 24 h after the return of spontaneous circulation (ROSC) [22]. In the only RCT on this subject, patients resuscitated from witnessed out-of-hospital ventricular fibrillation (VF) were randomized after the ROSC to be ventilated either with 30% or 100% oxygen for 60 min. Main outcome measures included NSE and S-100 protein concentrations at 24 and 48 h after ROSC, but unfortunately the study was not sufficiently powerful to show an effect on the brain injury markers [23]. Interventional studies are needed to determine the optimal oxygen concentration after cardiac arrest and the potential association between oxygen therapy and outcomes.

Arterial hypotension is common among patients resuscitated from cardiac arrest and it is associated with increased mortality [24]. Hemodynamic instability during the post-resuscitation period is thought to be caused by the severe, global ischemia-reperfusion injury associated with myocardial stunning [25], profound systemic inflammation [26], and adrenal axis suppression [27]. The autoregulation of CBF is disturbed and cerebral perfusion may become directly dependent on cerebral perfusion pressure (CPP), which is dependent on MAP [6]. The optimal MAP level after cardiac arrest is unknown and it is unclear whether the body’s intrinsic ability to maintain adequate perfusion pressure is just a predictor of good neurological recovery or whether supporting the circulation with vasoactive agents improves outcomes. In animal models, inducing hypertension with vasoactive agents during the post-resuscitation period has been related to less-severe brain injury and better neurological outcomes after asphyxial cardiac arrest [28]. In contrast, an observational human study of 168 patients found that vasoactive agent use in cases of post-cardiac arrest syndrome was associated with increased mortality and poor neurological outcomes [29]. According to another observational study, during the post-resuscitation period, MAP levels > 70 mmHg were associated with improved neurological outcomes, but if the patients needed vasopressor support to achieve this goal, there was no difference in recovery compared with patients with lower MAP levels and no use of vasopressors [30]. In a recent multi-center observational study that involved the high-frequency capture of MAP and vasopressor data, hypotension during the first 6 h after ICU admission was an independent predictor of worsened recovery after OHCA, but vasopressor load was not associated with poor outcomes [31]. RCTs are needed to evaluate whether interventions to support blood pressure improve neurological outcomes.

NSE is a cytoplasmic glycolytic enzyme specific to neurons and neuroectodermal cells. After neuronal damage, NSE is released into the cerebrospinal fluid and blood stream. Elevated or increasing levels during the first 24–72 h after cardiac arrest predict poor outcome [32, 33]. S100 is a neuroglial cell protein that is released into blood after cerebral damage and, as with NSE, elevated levels during the first 24–72 h after cardiac arrest predict poor outcome [32, 34]. Near-infrared spectroscopy (NIRS) is a non-invasive method to determine regional cerebral oxygen saturation (rSO_2_) and previous studies indicate its limited potential for early prediction of neurological outcome after cardiac arrest [3]. In the future, NIRS might be used as a continuous monitor to optimize cerebral perfusion and oxygenation after cardiac arrest, but further studies are needed. Changes in electroencephalogram (EEG) can also be used in prognostication after resuscitation. Presence of burst-suppression, status epilepticus, or poor reactivity on EEG after cardiac arrest and rewarming from therapeutic hypothermia are all related with poor neurological outcome [35].

According to current literature, high-normal PaCO_2_, PaO_2_, and MAP might contribute to lower levels of NSE and S100 and better neurological outcome after OHCA. With unconscious, mechanically ventilated patients, targeting specific levels of PaCO_2_, PaO_2_, and MAP should be feasible. The effect of different levels of PaCO_2_, PaO_2_, and MAP on NIRS and EEG during the post-resuscitation care is unknown. This article describes the study protocol for a prospective, multi-center, randomized pilot trial of targeting low- or high-normal carbon dioxide, oxygen, and mean arterial pressure after cardiac arrest and resuscitation (COMACARE).

## Methods/Design

The COMACARE trial is a prospective, multi-center, pilot RCT using 2^3^ factorial design. The main objectives of the study are to assess the feasibility of targeting low- or high-normal PaCO_2_, PaO_2_, and MAP in comatose, mechanically ventilated patients after OHCA and to evaluate the effect of low- or high-normal PaCO_2_, PaO_2_, and MAP on brain injury markers at 48 h after cardiac arrest. Secondary objectives are to assess the effect of low- or high-normal PaCO_2_, PaO_2_, and MAP after OHCA on cerebral oxygenation, epileptic activity, and neurological outcome six months after cardiac arrest. COMACARE is a RCT comparing multiple treatment groups with each other. There is no additional control group not receiving any study interventions. The trial is unblinded; due to the nature of the interventions, the treating personnel cannot be blinded regarding study group allocations.

### Outcomes

#### Primary outcome

- NSE serum concentration at 48 h after cardiac arrest

#### Secondary outcomes

- NSE serum concentration at 24 and 72 h after cardiac arrest
- S100 protein serum concentration at 24, 48, and 72 h after cardiac arrest
- Cardiac troponin (TnT) concentration at 24, 48, and 72 h after cardiac arrest
- Results of NIRS monitoring during the first 48 h after admission to the ICU
- Results of continuous EEG monitoring for 48 h after arrival at the ICU and a statement of the findings by an experienced senior neurologist/neurophysiologist
- Cerebral performance category (CPC) at six months after cardiac arrest
- Total duration of intensive care
- Total duration of mechanical ventilation
- Length of hospital stay
- Discharge destination
- Vital status at hospital discharge (dead or alive)

#### Feasibility outcomes

- Difference in PaCO2 between groups targeting low-normal (4.5-4.7 kPa) and high-normal (5.8–6.0) PaCO_2_
- Difference in PaO2 between groups targeting low-normal (10–15 kPa) and high-normal (20–25 kPa) PaO_2_
- Difference in MAP between groups targeting low-normal (65–75 mmHg) and high-normal (80–100 mmHg) MAP
- Distribution of values for primary and secondary outcomes
- Randomized/screened patient ratio
- Consent rate
- Data completion rate
- Recruitment duration

### Study setting and participants

Six intensive care units (ICUs) across Finland and one center in Denmark will participate in this trial. The participating Finnish hospitals are Helsinki University Hospital (Helsinki), Päijät-Häme Central Hospital (Lahti), Tampere University Hospital (Tampere), Central Finland Central Hospital (Jyväskylä), Kuopio University Hospital (Kuopio), and North Carelia Central Hospital (Joensuu). The Danish center is Aarhus University Hospital in Aarhus, Denmark. All patients admitted to one of the participating ICUs who experience ROSC after OHCA will be screened for eligibility.

### Inclusion criteria

- Witnessed OHCA with VF or ventricular tachycardia (VT) as the initial rhythm
- ROSC within 10–45 min from the start of arrest
- Arrest has confirmed or suspected cardiac origin
- Mechanical ventilation upon arrival at ICU
- Markedly impaired level of consciousness defined as no response to verbal commands and Glasgow Coma Scale (GCS) motor score < 5 (withdrawal to painful stimuli at best)
- Deferred consent possible or likely, or informed consent obtained, according to local ethical praxis
- Active intensive care initiated, including targeted temperature management (33–36 °C)

### Exclusion criteria

- In-hospital cardiac arrest
- Cardiac arrest with non-shockable initial rhythm (asystole or pulseless electrical activity)
- Arrest with a confirmed or presumed non-cardiac etiology
- Probable withdrawal from active ICU care due to terminal illness or poor prognosis because of severely reduced functional status before cardiac arrest
- Deferred consent impossible or unlikely (no known next of kin or relatives)
- Conscious patient or only mild impairment of consciousness (responsive to verbal commands or GCS motor score ≥ 5)
- Confirmed or suspected acute or pre-existing intracranial pathology and/or suspicion of increased intracranial pressure
- Age < 18 or > 80 years
- Pregnancy
- Severe oxygenation disorder (PaO_2_/FiO_2_ < 100 mmHg)
- Severe chronic obstructive pulmonary disease (COPD)

### Randomization and intervention

**Fig. 1:**
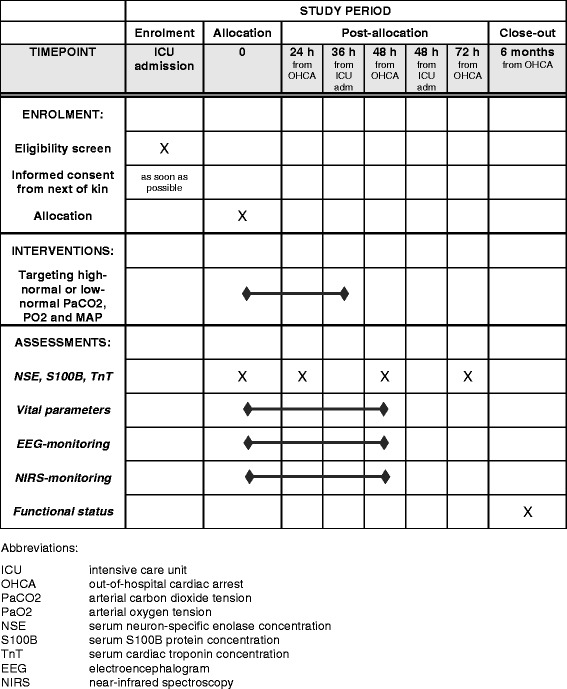
Schedule of enrolment, interventions, and assessments of the COMACARE trial

Schedule of enrolment, interventions, and assessments of the COMACARE trial are presented in Fig. 1. After ICU admission, participants are randomized into one of eight arms, with each arm having a different combination of PaCO_2_, PaO_2_, and MAP targets according to the 2^3^ factorial design:

1. Low-normal PaCO_2_ (4.5–4.7 kPa), low-normal PaO_2_ (10–15 kPa), low-normal MAP (65–75 mmHg);
2. High-normal PaCO_2_ (5.8–6.0 kPa), low-normal PaO_2_ (10–15 kPa), low-normal MAP (65–75 mmHg);
3. Low-normal PaCO_2_ (4.5–4.7 kPa), high-normal PaO_2_ (20–25 kPa), low-normal MAP (65–75 mmHg);
4. High-normal PaCO_2_ (5.8–6.0 kPa), high-normal PaO_2_ (20–25 kPa), low-normal MAP (65–75 mmHg);
5. Low-normal PaCO_2_ (4.5–4.7 kPa), low-normal PaO_2_ (10–15 kPa), high-normal MAP (80–100 mmHg);
6. High-normal PaCO_2_ (5.8–6.0 kPa), low-normal PaO_2_ (10–15 kPa), high-normal MAP (80–100 mmHg);
7. Low-normal PaCO_2_ (4.5–4.7 kPa), high-normal PaO_2_ (20–25 kPa), high-normal MAP (80–100 mmHg);
8. High-normal PaCO_2_ (5.8–6 kPa), high-normal PaO_2_ (20–25 kPa), high-normal MAP (80–100 mmHg).

Study centers will have access to a web-based randomization system. A cryptographically strong random number generator with modulo bias eliminated is used to generate random numbers and an unbiased Fisher-Yates (Durstenfeld) algorithm is used to shuffle blocks. Randomization is stratified with respect to target temperature (33 °C or 36 °C).

**Fig. 2:**
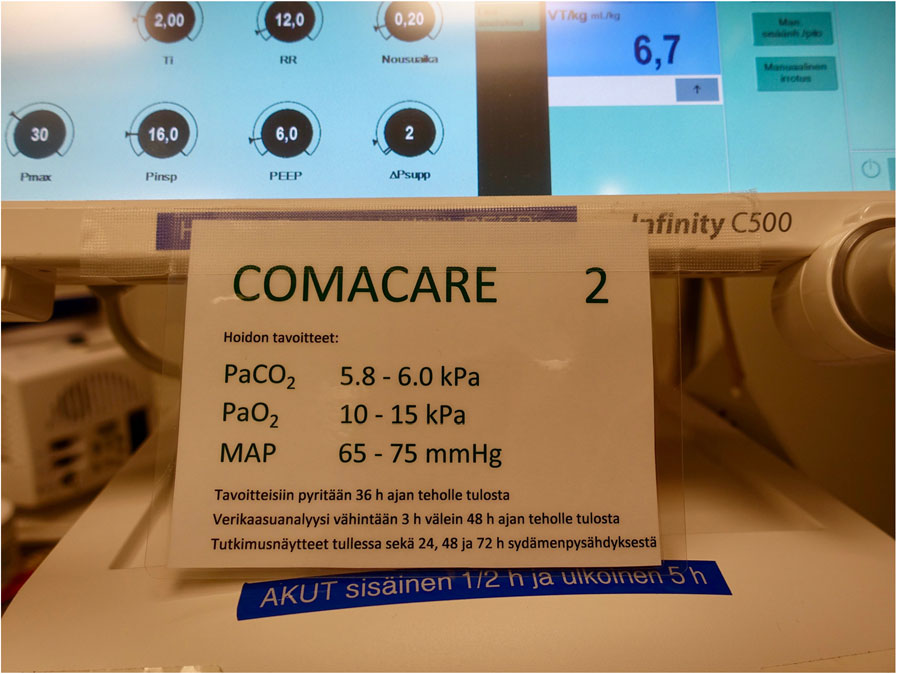
Picture of the COMACARE treatment target card attached to a ventilator

The treating clinicians and nurses will be made aware of treatment targets using laminated signs at the patient’s bedside and on mechanical ventilators (Fig. 2).

Low-normal and high-normal carbon dioxide levels will be targeted by adjusting minute ventilation (tidal volume and frequency) based on the results of an ABG analysis (corrected to the patient’s actual temperature) and using the corresponding end-tidal carbon dioxide (EtCO_2_) value as an additional guide. Low-normal and high-normal PaO_2_ levels will be targeted by adjusting the fraction of inspired oxygen (FiO_2_) in the range of 21–100% and using moderate levels (8–10 cmH_2_O) of positive end-expiratory pressure (PEEP). Peripheral oxygen saturation (SpO_2_) can be used as an additional guide in the low-normal PaO_2_ group in order to avoid hypoxia and to remain within the target range. In the high-normal PaO_2_ group, SpO_2_ will be close to 100% and it cannot be used to monitor the oxygenation reliably. Arterial blood gas samples will be obtained at least every 3 h to ensure that blood gas levels are within the target ranges. Low-normal and high-normal MAP levels will be targeted by using a continuous infusion of noradrenaline as needed. Fluid boluses are allowed for the treatment of hypovolemia, according to the treating clinician’s preference. In cases of confirmed or suspected low cardiac output, the use of an inotrope, such as dobutamine or levosimendan, is allowed. There are no limits to the infusion rates of noradrenalin or inotropes and the dose will be determined according to local ICU protocols and the treating clinician’s preference. No efforts other than sedation and pain medication will be made to lower blood pressure levels in order to meet the low-normal target. In cases of hypertensive crisis (defined as MAP > 140 mmHg) or suspected or detected left ventricular systolic dysfunction, the blood pressure may be lowered using vasodilating agents, according to the treating clinician’s preference.

No further instructions of how to achieve the target levels of PaCO_2_, PaO_2_, and MAP will be given to the treating clinicians. The intervention will continue for 36 h or until the patient is extubated or ventilation is set to a spontaneous mode.

### Sample size

For the power analysis, we assumed that there is no interaction between PaCO_2_, PaO_2_, and MAP and considered each intervention independently. The primary endpoint will be the NSE value at 48 h from the arrest. Based on our previous cohort of OHCA patients, the mean NSE at 48 h is around 17 μg/L and the standard deviation is around 20 [36]. In a previous, small RCT, the use of 30% FiO_2_, compared with 100% FiO_2_, resulted in approximately 50% increase of NSE values at 48 h in the subset of patients treated with hypothermia [23]. Assuming this previous finding, a study with 39 patients in each arm would have a power of 80%, with the significance set at 0.05, to detect a 50% increase in NSE. Given the possibility of death prior to 48 h and loss of follow-up, we will include 50% more patients than the target. Therefore, a total of 120 patients will be included (60 patients in each intervention arm).

### Data collection

Basic information regarding participants’ age, gender, prior health status, and functional capacity and the details of the resuscitation procedure will be saved in a web-based study database. Participants will be subjected to standard monitoring in the ICU. All monitored vital parameters will be saved using data collection software (S/5 Collect version 4.0, GE Healthcare, Helsinki, Finland) and a medical-approved tablet computer (Arbor M1040, Taiwan) connected to the monitor for 48 h after ICU admission.

During the first 48 h after ICU admission, ventilation settings will be obtained directly from the ventilator and saved in the study database. The doses of any sedative and vasoactive drug infusions, ABG analysis results, and other laboratory test results for the 48 h after ICU admission will be manually entered into and saved in the study database.

**Fig. 3:**
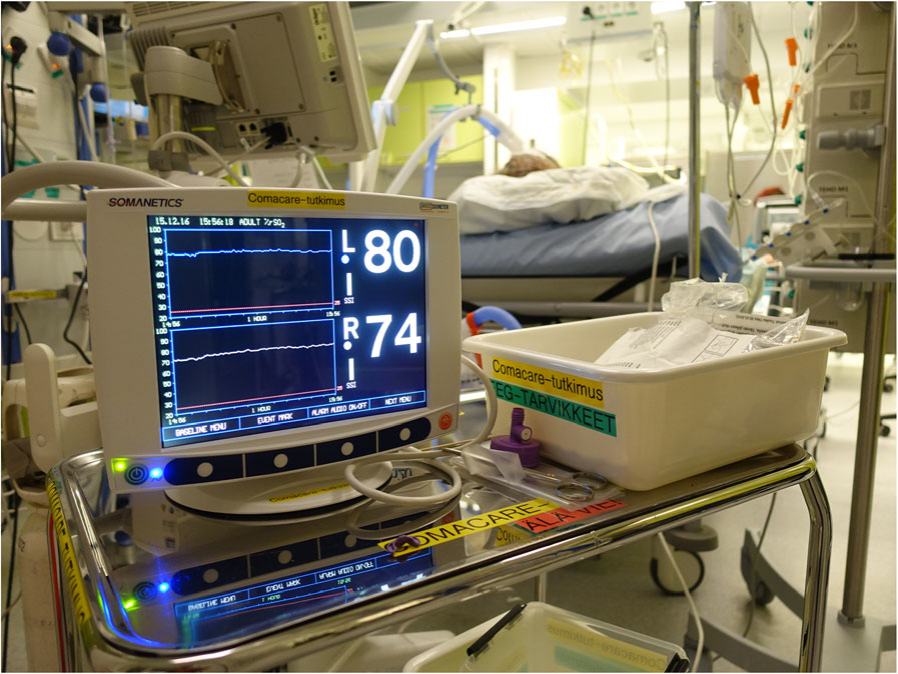
Picture of the near-infrared spectroscopy monitor blinded from the treating personnel

Regional cerebral oxygen saturation will be measured and stored continuously for 48 h after ICU admission using a non-invasive NIRS device (Covidien INVOS™ Regional Oximetry). The treating clinicians and nursing staff will be blinded to the NIRS monitoring (Fig. 3).

Continuous four-channel EEG monitoring is applied upon admission to the ICU and recorded for 48 h after admission. The EEG recordings are collected and analyzed offline by a neurophysiologist. These EEG recordings will be categorized into three groups according to the degree of abnormality, specifically into the mild, moderate, and severe categories proposed by Crepeau et al. [37]. The doses of sedative agents (propofol, midazolam, and opioids) will be recorded in the study database. All patients undergo targeted temperature management (TTM) treatment and will receive continuous sedation according to local protocol. The study is stratified by site and we do not anticipate significant difference in the doses of sedative agents between intervention groups. However, we will report any possible differences in the amount of sedation administered between groups. The EEG analysis will also include the deepness of anesthesia (inactivity, burst-suppression, continuous slow/fast, reactive). Epileptic bursting activity in the EEG is also measured, particularly during weaning from anesthesia. Epileptic EEG activity is defined as general, lateralized, or focal bursts of spiking characterized by changes in frequency and/or location. High- or low-frequency generalized periodic discharging (GPD) in the EEG is classified as an electrophysiological sign of encephalopathy.

Additional blood samples will be obtained upon ICU admission and at 24, 48, and 72 h after cardiac arrest. The samples will be centrifuged and frozen at – 70 °C for the later analysis of serum NSE, S100b protein, and cardiac troponin concentrations or, if this is not logistically possible, for the immediate analysis of NSE, s100b and cardiac troponin using the same laboratory assays. A neurologist blinded to the study group allocations will contact all hospital survivors or their surrogates by telephone six months after cardiac arrest to determine the patients’ functional statuses using the cerebral performance category (CPC) scale.

In addition, the patients will be subject to all standard monitoring and investigations as needed according to the local protocol of the ICU. This will include prognostic evaluation in case the patient does not recover after 36-48 h.

### Sub-study including magnetic resonance spectroscopy

For participants treated at Meilahti University Hospital, Helsinki, a magnetic resonance spectroscopy (MRS) scan will be performed 48–120 h after cardiac arrest to determine the acid-base status of the central nervous system. This is not routine practice for cardiac arrest patients; therefore, informed consent from the patient’s next of kin will be obtained before performing the MRS. The MRS scan will be performed while the patient is intubated and the patient will be monitored by a specialist in anesthesiology and a qualified ICU nurse.

Intracellular pH will be determined using ^31^P MR spectroscopy. A 6 × 6 × 6 cm^3^ voxel will be placed mainly in the parietal lobe overlapping with vascular territories of the anterior cerebral artery (ACA), middle cerebral artery (MCA), and posterior cerebral artery (PCA), utilizing T1-weighted localization images collected in three orthogonal planes. pH will be calculated by placing the chemical shift difference (δ) of Pi and PCr resonances to Hendersson–Hasselbach formula: pH = 6.77 + log{(δ-3.29)/(5.68-δ)} [38].

T2 relaxation time of water will be determined by ^1^H MRS collecting data with six different echo times (10, 25, 40, 55, 70, 85, and 100 ms) from a 15 × 15 × 15 mm^3^ voxel placed in centrum semiovale with a STEAM localization. Increased T2 serves as a marker of increased free water (edema).

### Statistical analysis

Categorical data will be presented as counts and percentages and compared with a Chi-square test. All continuous data will be checked for normality and presented as mean and standard deviation or medians with 25th and 75th quartile points. Data with a normal distribution will be compared with Student’s t-test and data with a non-normal distribution with the Mann–Whitney U test. Hourly median MAP values will be calculated from all stored MAP values and these values will be used in the analysis. Low-normal and high-normal PaCO_2_, PaO_2_, and MAP values will be compared over time using a generalized mixed model with a compound-symmetry covariance matrix. The mixed model warrants a near-normal distribution of sample and, therefore, if this is violated, logarithmic transformation will be performed and completed with a non-parametric pair-wise comparison. The values and area under the curve will be calculated for each patient and compared between the groups. In the corresponding low-normal and high-normal PaCO_2_, PaO_2_, and MAP groups, NSE levels will be compared separately at 48 h using the Mann–Whitney test. The NSE values over time will also be compared using a generalized mixed model. Kaplan–Meier curves will be constructed separately for each of the three groups to visualize survival over time. Logistic regression will be used only in case of a significant imbalance in the baseline factors, but conclusions will be based on univariate testing. The proportional distribution of EEG abnormality (categorized as mild, moderate, or severe for each patient) between treatment groups will be compared with a Chi-square test.

### Data and safety monitoring

Three experts on intensive care research will act as the data and safety monitoring board for the study. The predefined serious adverse events (SAE) that could be related to the treatment interventions include severe hypercapnia and respiratory acidosis (PaCO_2_ > 10 kPa and pH < 7.15), unexplained brain edema on CT scanning, and severe unexplained ARDS (PaO_2_/FiO_2_ ratio of < 100 mmHg). All these events will be recorded in the study database and reported to the study’s principal investigators within 24 h. If applicable, the study intervention will be interrupted immediately and the patient will be treated according to the standard protocol of the ICU.

## Discussion

Trial recruitment began in Meilahti Hospital, Helsinki, in March 2016. Five additional Finnish centers joined the trial between May and December 2016 and Aarhus University Hospital joined the trial in March 2017. Participant recruitment is expected to be completed by December 2017 and the six-month neurological assessment is expected to be completed by June 2018. The study will be published as three separate papers comparing the effects of carbon dioxide, oxygen, and MAP on the primary and secondary outcomes. We aim to publish all the studies at the same time in the same journal. The results of the COMACARE trial will provide preliminary clinical evidence regarding the feasibility of targeting low- or high-normal values of PaCO_2_, PaO_2_, and MAP in comatose patients after cardiac arrest and resuscitation. It will also provide preliminary information on the effect of low- or high-normal levels of PaCO_2_, PaO_2_, and MAP on neurological injury biomarker concentrations, brain oxygenation, and epileptic activity. The results of this trial will be used to evaluate whether a larger RCT on this subject that is sufficiently powerful to determine the effects of low- or high-normal levels of PaCO_2_, PaO_2_, and MAP on survival and neurological recovery at six months is justified (Additional file 1).

## Trial status

Recruitment is active in all six study sites in Finland and in one study site in Denmark. Currently, 115 patients have been included.

## Additional file

Additional file 1: Spirit checklist. Populated SPIRIT checklist for the COMACARE trial. (PDF 2736 kb)

## Abbreviations

CBF: Cerebral blood flow
EEG: Electroencephalogram
GCS: Glasgow coma scale
GPD: Generalized periodic discharging
ICU: Intensive care unit
MAP: Mean arterial pressure
NIRS: Near-infrared spectroscopy
NSE: Neuron-specific enolase
OHCA: Out-of-hospital cardiac arrest
PaCO_2_: Arterial carbon dioxide tension
PaO_2_: Arterial oxygen tension
PEEP: Positive end-expiratory pressure
ROSC: Return of spontaneous circulation
VF: Ventricular fibrillation
VT: Ventricular tachycardia
